# Adhesion prevention after endometriosis surgery — results of a randomized, controlled clinical trial with second-look laparoscopy

**DOI:** 10.1007/s00423-021-02193-x

**Published:** 2021-05-26

**Authors:** Bernhard Krämer, Jürgen Andress, Felix Neis, Sascha Hoffmann, Sara Brucker, Stefan Kommoss, Alice Höller

**Affiliations:** Department for Women’s Health, University Hospital Tübingen, Calwerstr. 7, 72076 Tübingen, Germany

**Keywords:** Clinical study, Deep infiltrating endometriosis, Second look, Adhesion prophylaxis, Barrier gel, 4DryField^®^ PH

## Abstract

**Purpose:**

Adhesion formation after endometriosis surgery is a severe problem affecting up to 90% of patients. Possible complications include chronic pain, ileus, and secondary infertility. Therefore, effective adhesion prophylaxis is desirable, for which the adhesion barrier 4DryField^®^ PH is evaluated in the present clinical study. It is a starch-based powder that forms a gel after irrigation with saline solution and thus separates surgical sites as physical barrier for adhesion prevention.

**Methods:**

Fifty patients with extensive and deep infiltrating endometriosis were included in this prospective, randomized, controlled clinical trial with two-staged laparoscopic approach. The patients were randomized into two groups, one receiving 4DryField^®^ PH and the other irrigation with saline solution for adhesion prevention. Adhesion formation was directly scored during second-look interventions considering incidence, extent, and severity. Adhesion prevention treatment in the second surgery was performed corresponding to the first intervention to evaluate the long-term outcome in the later course.

**Results:**

Both groups were comparable with respect to relevant patient parameters. Severity and extent of adhesions were significantly reduced by 85% in the 4DryField^®^ PH group compared to the control group (mean total adhesion score 2.2 vs. 14.2; p = 0.004). Incidence of adhesion formation based on the number of affected sites was significantly reduced by 53% in the intervention vs. control group (mean 1.1 vs. 2.3 sites; p = 0.004). Follow-up of secondary endpoints is not yet completed; results will become available at a later stage.

**Conclusion:**

Adhesion formation could be reduced significantly by 85% by application of the adhesion barrier 4DryField^®^ PH.

**Trial registration:**

Trial registration main ID: DRKS00014720, secondary ID: U1111-1213-4142; date of registration 09th May 2018.

## Introduction

Endometriosis affects between 10% and 15% of women of reproductive age [1]. The most common accessory symptoms are infertility and the so-called endometriosis-associated pelvic pain, a term that commonly includes dysmenorrhea, non-cyclical pelvic pain, deep dyspareunia, dyschezia, and chronic pelvic pain [2–4]. The pathogenesis is still poorly understood, assuming retrograde menstruation leading to the attachment and implantation of endometrial glands and stroma on the peritoneum as the most probable mechanism. Other theories suspect coelomic metaplasia and hematogenous or lymphatic spread [5, 6].

As no clear diagnostic serum markers have been identified to date, endometriosis can only be definitely diagnosed through a histopathological evaluation providing evidence of endometrial glands and stroma showing signs of inflammation and fibrosis [7].

The medical management of endometriosis is based on nonsteroidal anti-inflammatory drugs or endocrine treatment [7].

Despite the pharmaceutical options, treatment often requires surgery to excise or ablate the endometrial tissue [8], which can be associated with infections, bowel obstruction, diminished ovarian reserve, as well as the development of adhesions [9–11]. They develop in more than 90% of surgeries in the abdominal cavity and are therefore a major concern [12] due to their short- and long-term complications [13, 14]. Furthermore, adhesions may even play a role in the development of some forms of endometriosis such as ovarian endometriomas and deep invasive nodules [15].

Minimally invasive laparoscopic approaches can also lead to adhesions in > 80% of patients with severe endometriosis and complete posterior cul-de-sac obliteration [16]. In cases with resection of stages I–III endometriosis, adnexal AFS adhesion score increased from 10 to 14, correlating with the baseline endometriosis stage. Additionally, in the presence of at least 50% of red endometriotic lesions, there was a greater score increase than in patients with mostly black or white and/or clear lesions [17]. In a study on the outcome of adhesiolysis surgery, the presence of endometriosis led to inferior adhesion reduction [18].

Consequently, effective adhesion prevention after minimally invasive endometriosis surgery is highly desirable and several attempts have been made to introduce specific barrier agents into clinical routine. Laparoscopy appears to reduce adhesiogenesis [19], but reformation is also common here [20, 21] and no barrier agent produced comprehensive satisfactory results [22] or reliable superiority in comparative trials to date.

In this study, 4DryField^®^ PH, a novel adhesion barrier, was investigated for adhesion prevention after endometriosis resection in a second-look design. It is a powder based on purified potato starch that transforms into a gel with saline solution. The gel then acts as a temporary physical barrier between the surgically traumatized peritoneal surfaces until mesothelial healing is completed and it is subsequently resorbed. Observational and retrospective trials from visceral [23, 24] and gynecologic surgery (including endometriosis patients) [25, 26] showed promising results, which are to be verified in this first randomized controlled trial (RCT).

## Material and methods

### Patient collective

This RCT was approved by the Ethics Committee of the Medical Faculty at our institution (no. 217/2018BO1). It is registered in the German Clinical Trial Register (DRKS) and the International Clinical Trials Registry Platform of the World Health Organization (main ID: DRKS00014720, secondary ID: U1111-1213-4142). Informed consent was obtained from all patients included. Fifty women with laparoscopic resection of endometriosis in a two-step approach were randomized into two groups between July 2018 and November 2019. All surgeries and follow-up visits were conducted at the Department for Women’s Health at the University Hospital Tübingen. As there is no placebo for the tested medical device, only the patients were blinded.

The relevant inclusion criteria were histological diagnosis of deep infiltrating endometriosis (DIE) or extensive peritoneal and/or ovarian endometriosis upon diagnostic first-look laparoscopy with the indication for a definite subsequent therapeutic procedure according to our center’s practice (second-look laparoscopy). Pregnant and/or breastfeeding patients, patients with known incompatibility of starch-containing substances, and patients without resection of endometrial tissue for histological confirmation during the first laparoscopy, and therefore not requiring any adhesion prevention treatment, were excluded.

The patient collectives of the two study arms were statistically tested for comparability regarding the following parameters: age, BMI, duration of first surgery, frequency of outpatient treatment (1st surgery), previous endometriosis surgeries, metabolic disorders, severity of pre-operative pain (on a scale from 0 to 10), and rASRM and ENZIAN endometriosis scores. ENZIAN scores were compared based on arithmetic means calculated for the subscores A, B, and C as their severity is assessed with a numerical value, whereas frequencies were compared for the F-subscores.

### Study process and general minimally invasive approach

**Fig. 1:**
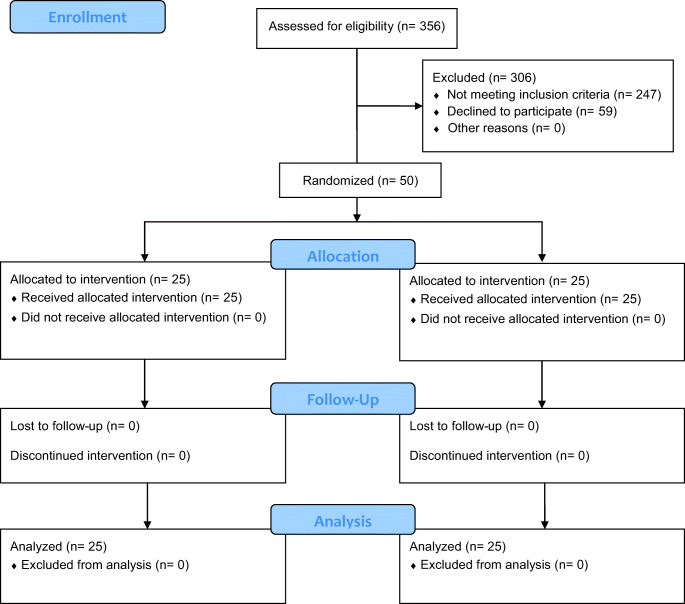
Flowchart describing the study process

The flowchart in Fig. 1 describes the study process. The follow-up that Fig. 1 refers to only includes adhesion scoring during the second surgery, not follow-up after that to collect secondary endpoint data. All the 247 patients that did not meet the inclusion criteria had their endometriosis completely resected during the initial surgery and therefore did not fulfil the criterion of an indication for a definite subsequent therapeutic procedure. The trial was designed as a parallel study with an allocation ratio of 1. Randomization was performed using simple randomization utilizing a randomization list generated using the RAND function of Microsoft Excel. The randomization list was generated by the study nurse who was the only person unblinded to randomization. She was present at all first surgeries as randomization was performed during surgery. Participants were only enrolled by surgeons participating in the study.

During the first intervention, we aimed to excise endometriosis both for histological confirmation and for symptom relief. Accordingly, in some cases, several sites (endometriotic lesions and/or adhesions) that were considered relevant were treated, even if the patient would not undergo the second operation for the complete excision of deeper infiltrating lesions. For moderate lesions with only peritoneal or superficial endometriosis, resection was performed exclusively during this intervention and the patient did not require a second intervention, resulting in exclusion from the study.

In contrast, if additional DIE or extensive peritoneal or ovarian endometriosis (e.g., kissing ovaries) were present upon first-look laparoscopy, endometriosis was excised locally for histological confirmation and a second surgery for definite treatment was planned. The study nurse only pronounced group assignment afterwards and an anti-adhesive treatment at the excision sites followed according to randomization. This two-stage concept for advanced endometriosis represents the practical routine in our center and allows optimized interdisciplinary planning with respect to the extent of the condition, as well as to the patient’s leading symptoms (pain, fertility issues, organ obstruction, etc.).

After 3–16 weeks, the second intervention with the definitive therapeutical resection of DIE, optionally with partial removal of involved organs such as ureter, bowel, or bladder, as well as the excision of remaining peritoneal or ovarian endometriosis, was carried out. The timing of the second intervention was based on the routine interval for this surgery in our hospital. It takes into account an adequate recovery time after the first intervention and allows for completion of possible adhesion reformation for lysis during the second operation. The time interval was also chosen for optimal conditions for the second surgery. This included the peritoneal healing of the defects, as well as optimized planning and consent in cases of higher risk resections of deep infiltrating endometriosis adjacent to the ureter/bowel or even bowel interventions (shaving, disc, or full thickness resection). A few cases were delayed due to patients’ personal reasons. In the intervention group, the mean interval between both interventions was 8.1 weeks (range 5–16 weeks), in the control group 7.4 weeks (2.9–15.6 weeks). During the second intervention, the previously resected and treated locations (first laparoscopy) with potential subsequent adhesion formation were investigated. All 50 patients that were randomized also had their second intervention to complete endometriosis resection and were therefore included in the primary outcome evaluation.

**Fig. 2:**
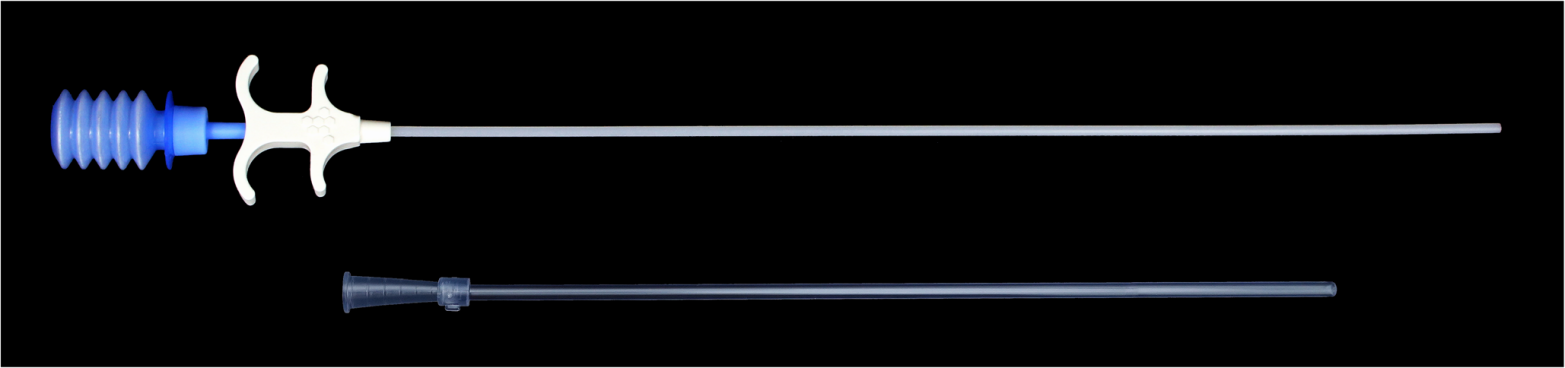
The laparoscopic application device 4DFLap^TM^ connected to the bellow bottle applicator containing 4DryField^®^ PH

In the intervention group, 4DryField^®^ PH was applied for adhesion prevention in both surgeries. It was applied at the end of each intervention as a powder until all surgically affected areas were completely covered and then transformed into a gel by irrigation with saline solution. The mean amounts used were 3.2 g powder (range: 1–5 g) and 10.5 ml saline solution (range: 2–20 ml). The powder was applied through the corresponding laparoscopic applicator 4DFLap™ (Fig. 2), saline solution was administered via a standard laparoscopic irrigation instrument for controlled flushing until the gel was formed.

In the control group, only saline solution was used for flushing with a mean amount of 40.9 ml (range: 5–200 ml).

### Assessment of adhesion *extent* and *severity*

Adhesions were assessed during both interventions with a score modified from the American Fertility Society (AFS) score [27]. In both surgeries, sixteen previously defined specific areas of interest were evaluated: right ovary, left ovary, uterosacral ligament, round ligament of the uterus, ovarian fossa, right fallopian tube and broad ligament of the uterus, left fallopian tube and broad ligament of the uterus, uterine serosa, rectum surface, sigmoid colon surface, coecal pole, vagina, pouch of Douglas, psoas region, pelvic diaphragm, and rectovaginal septum.

During the first surgery, *the extent* of possible adhesion formation sites (predilection sites) resulting from the planned endometriosis resection and/or from intended adhesiolysis was rated on a scale from 0 to 4 (0: not affected, 1: localized (less than 1/4 of the area affected), 2: moderate (between 1/4 and 2/4 of the area affected), 3: pronounced (between 2/4 and 3/4 of the area affected), 4: extensive (more than 3/4 of the area affected)).

During the second surgery, the same sixteen regions were assessed for post-operative adhesions and their extent was scored in accordance with the first surgery. Additionally, *the severity* was scored as either 0, 1, or 4 (0: no adhesions, 1: mild (thin, avascular), 4: severe (dense, vascular)). Corresponding to the AFS score, severity and extent were then multiplied to yield a site score. The sum of all site scores added up to the total score:

$$ Totalscore=\sum \limits_{allsites}\left( extentscore\bullet severityscore\right) $$

The score used in the present study deviates from the AFS score in the evaluation of sixteen (instead of four) areas and a finer extent score (scores 0, 1, 2, 3, 4 instead of 0, 1, 2, 4).

### Secondary outcomes

*The incidence* of adhesion formation upon second look is given as the number of sites that were affected after the first intervention.

Secondary endpoints to evaluate during follow-up include post-interventional pain (non-cycle dependent, dysmenorrhea, dyspareunia, dyschezia, dysuria) and the number of complications (impaired wound healing, infections), necessary re-operations for adhesiolysis and for other reasons, as well as pregnancies. Data for the secondary endpoints will be collected during the 12-month follow-up after the second intervention and will become available at a later stage. In the present paper, only complications that developed in the study interval between the first surgery and second look are included.

### Statistics

Sample size determination was performed using G*Power 3.1 [28]. Results published by Korell et al. [26] and DiZeraga et al. [17] were used for this. Based on these, for the control group, it was assumed that 43.75% of the maximum possible adhesion score would be reached (SD: 9.375%) and for the 4DryField group 25.0% (SD: 35.35%); published scores were 14.0 on a scale from 0 to 32 for the control and 0.5 on a scale from 0 to 2 for the intervention group. Combined with a one-sided p-value of 0.05, a statistical power of 0.8, and an allocation rate of 1, the calculation led to a required sample size of 50.

The statistical evaluation was performed using Prism 7 for Windows (GraphPad Software Inc.). Continuous variables were tested for normality of distribution with the D’Agostino-Pearson test. If normally distributed, unpaired t-tests and if not, Mann-Whitney tests were employed (both always two-tailed). Categorical variables were evaluated using Fisher’s exact test and time-to-event variables using the Mantel-Cox test. Level of significance was always 0.05.

For calculation of the effect sizes, Glass’ ∆ was used when the variances were significantly different (determined by F-test) and Cohen’s d when they were not.

## Results

All outcomes presented result from analyzing all the 25 patients of the respective groups, none of which were reassigned.

### Basic parameters

The basic parameters are summarized in Table 1. No significant differences between the two groups were found regarding any of the examined values.

**Table 1:** Comparison of mean basic patient parameters (with standard deviations) for the two groups

		Intervention group	Control group	p
n		25	25	
Age [a]		29.7 ± 5.7	31.9 ± 6.2	0.194
BMI		23.5 ± 4.2	24.1 ± 3.8	0.593
Duration 1st surgery [min]		38.7 ± 14.6	43.7 ± 16.3	0.292
Previous endometriosis surgery		20%	24%	> 0.999
Ambulatory/outpatient setting		88%	96%	0.349
Metabolic disorder		0%	0%	> 0.999
rASRM score		2.2 ± 0.7	2.0 ± 0.7	0.459
ENZIAN scores	A	0.4 ± 0.8	0.4 ± 0.6	0.815
	B	1.4 ± 0.8	1.5 ± 0.9	0.716
	C	0.1 ± 0.5	0.1 ± 0.3	> 0.999
	FA	22%	35%	0.514
	FB	13%	26%	0.460
	FU	0%	0%	> 0.999
	FI	0%	0%	> 0.999
	FO	0%	0%	> 0.999
Pain scores (0–10)	Non-cycle dependent pelvic pain	4.6 ± 3.2	4.6 ± 3.5	0.984
	Dysmenorrhea	8.2 ± 2.2	7.6 ± 2.5	0.320
	Dyspareunia	3.0 ± 3.0	3.3 ± 3.4	0.769
	Dyschezia	2.0 ± 3.0	1.7 ± 3.0	0.735
	Dysuria	1.5 ± 2.4	0.6 ± 1.6	0.087

### Adhesion formation

The parameters *extent and severity* of adhesions as well as the adhesion *incidence* were analyzed in this clinical study.

*Severity and extent* of adhesions that could be detected upon second look are summarized in the total adhesion score according to the calculation described above. The total adhesion score for the control group is 14.2 (SD: 18.9). In the intervention group, the score is 2.2 (SD: 3.1). This represents a reduction of 85%. The difference is statistically significant (p = 0.004). The effect size (Glass’ ∆) is 0.64.

**Fig. 3:**
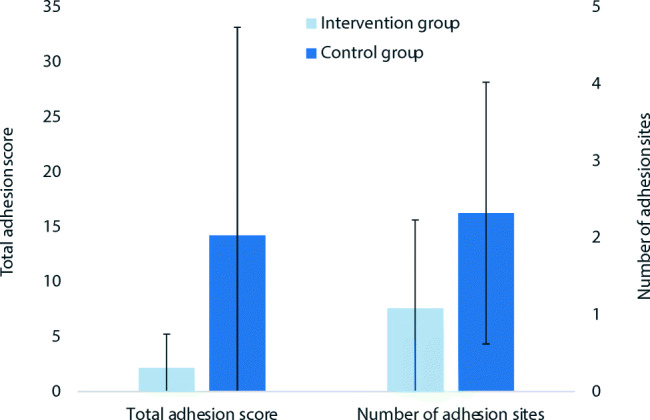
Total adhesion score and adhesion incidence (as number of adhesion sites) for the two groups (with standard deviations as error bars). Both differences are statistically significant (p < 0.05)

**Fig. 4:**
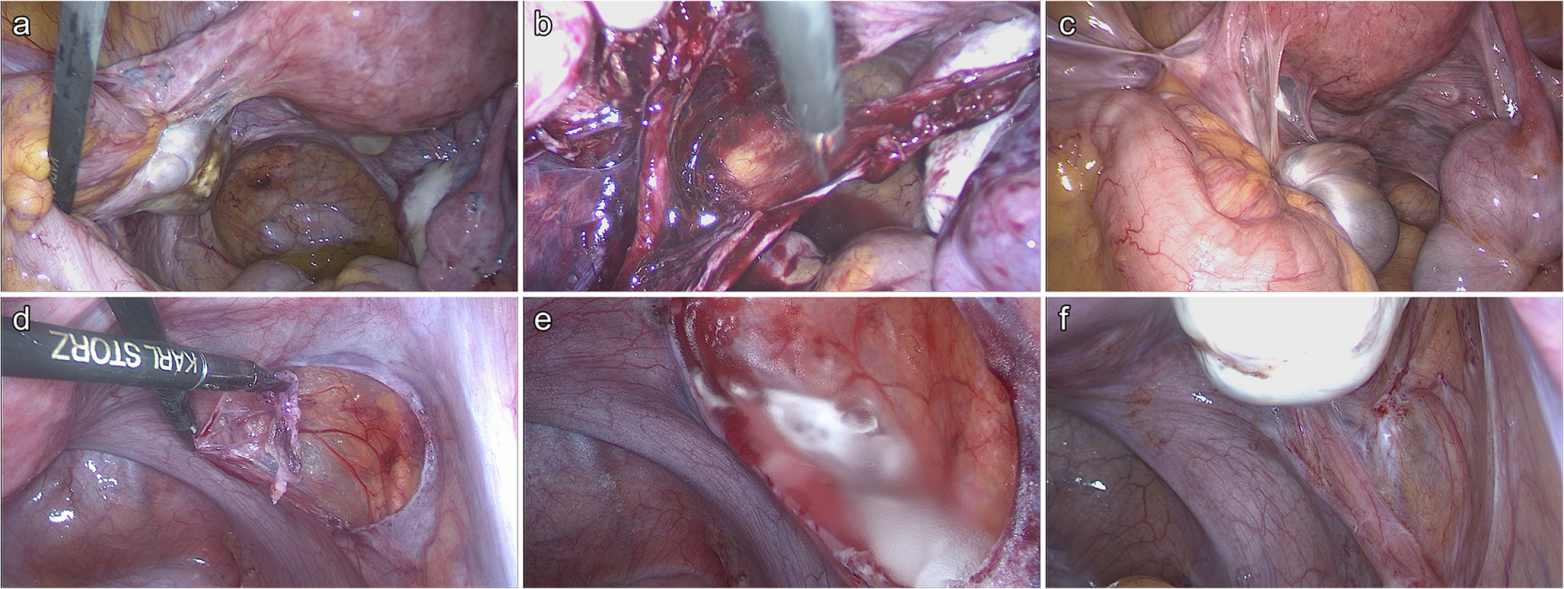
Representative photographs of predilection sites and adhesions from the two groups. **a**, **b** first and **c** second interventions of the same patient from the control group: **a** initial situs, **b** situs after adhesiolysis/endometriosis resection and before application of saline solution, **c** situs at second look. **d**, **e** first and **f** second interventions of the same patient from the intervention group: **d** situs during resection of endometriosis, **e** situs showing the 4DryField^®^ PH adhesion barrier after gel transformation, **f** situs at second look

In the control group, the mean incidence is 2.3 sites (SD: 1.7) and in the intervention group 1.1 (SD: 1.2), which is equivalent to a reduction of 53%. This difference is also statistically significant (p = 0.004). The effect size (Cohen’s d) is 0.87. The results are summarized in Fig. 3 and representative photos of predilection sites and adhesions from the two groups are displayed in Fig. 4.

### Secondary outcomes

So far, none of the 50 patients had infections or wound problems. No conclusive data for other secondary outcomes has been analyzed yet, but will become available at a later stage after the completion of follow-up.

### Harm

No harm or unintended effects resulting from study-specific treatment could be observed.

## Discussion

Even though various anti-adhesion agents or barriers have been shown to be effective in experimental or even human trials, they are still not used regularly in clinical practice. Apart from economic issues where reimbursement is not clearly defined in some health systems, the most important scientific reason is the lack of clinical studies with a well-designed second-look setting that produce reliable and comparative data regarding efficacy and better outcomes of competitive agents. Clearly, such evidence-based results would encourage and legitimate the use of the most efficacious products in clinical routine. The ideal adhesion barrier is beneficial, degradable in the human organism, can be applied laparoscopically, and is cost-effective.

Because of the mentioned methodical problems, this human study was designed to prospectively evaluate the anti-adhesive qualities of 4DryField^®^ PH vs. a control group *in a second-look approach*.

The results show a distinct and statistically significant reduction of adhesion extent, severity, and incidence after the application of the tested agent in comparison to irrigation with saline solution (control) only. The calculated effect sizes show a medium to large effect for the reduction of the adhesion score and a large effect for the reduction of adhesion sites. The analyzed reduction of the adhesion score of 85% is in line with previously published results from experimental animal trials, where the product achieved reductions of the adhesion score ranging from 85 to 100% [29–31]. Previous evaluations were designed with second looks, but the patient collectives were smaller, and a retrospective protocol was used. Nevertheless, these trials already suggested good adhesion prevention capabilities [23–26]. Pursuing these preliminary findings, our manuscript represents the first prospective and randomized clinical study to evaluate and compare the adhesion reduction of this agent.

Adhesion formation highly depends on the peritoneal trauma caused during an intervention. Therefore, extent and incidence of trauma sites (referred to as predilection sites in our study) created in the initial surgery (first intervention) during endometriosis resection or adhesiolysis were evaluated as well. Both were higher in the intervention group, with a mean total extent score for the predilection sites of 4.1 (SD: 3.9) vs. 2.4 (SD: 1.7) in the control groups and a mean number of predilection sites of 2.5 (SD: 1.4) vs. 1.4 (SD: 0.7). Both differences are significant (p = 0.025 and 0.003, respectively). This must be taken into consideration, as the statistically significant reduction of adhesion severity, extent, and incidence after 4DryField^®^ PH application, therefore, is unlikely to have been caused by more extensive first excisions in the control group.

Comparable studies for adhesion prevention after endometriosis resection are scarce; only five RCTs with second look have been published yet [32]: Mais et al. [16] used Interceed^®^, an oxidized regenerated cellulose-based barrier from Johnson & Johnson Medical Inc., in 16 women after laparoscopic endometriosis surgery and compared them to an equally sized, untreated control group. They only evaluated the adhesion incidence, which could be reduced by 63%. More detailed results regarding adhesion extent and severity were not reported in this publication. Sekiba et al. [18] also used Interceed and an internal control design where one pelvic sidewall was covered with the adhesion barrier and one was left uncovered in each of the 28 endometriosis patients. They did not report adhesions scores, but the number of sidewalls with adhesions. These were 23 in the control and 14 in the control group, a statistically significant improvement of 32%. Interceed was also used by Wallwiener et al. [33], who included 40 patients and classified the adhesions on a scale from 0 to 3. The resulting mean scores were 0.4 in the intervention and 1.1 in the control group, a difference that was not statistically significant. DiZerega et al. [17] investigated the use of Oxiplex/AP^®^, a gel based on polyethylene oxide and sodium carboxymethylcellulose from FzioMed Inc., in 20 patients after laparoscopic surgical treatment of endometriosis and compared them to an untreated control group of 17 patients. They used the adnexal adhesion scoring system according to the AFS, which delivered values of 14.0 for the controls and 6.2 for the intervention group, a reduction of 56%. However, the scores of both groups at first surgery differed by 1.6 to the disadvantage of the control group, meaning that control patients already had higher adhesion scores at first interventions. Brown et al. [34] used Adept^®^, an icodextrin solution from Baxter Healthcare, for adhesion prevention after laparoscopic gynecological surgery and compared it to Ringer’s lactate solution. The study included a subpopulation of endometriosis patients (122 in the intervention and 119 in the control group). They used the AFS score for the assessment of adhesion severity and extent but did not report the values; they only stated the percentage of patients with clinical success (which they defined as “the percentage of patients in whom the number of sites with adhesions decreased by at least 3 or 30% of the number of sites lyzed”). This success rate was 42% in the Adept^®^ and 37% in the control groups. None of these studies included secondary outcomes like pain or fertility development. In a Cochrane database review comprising adhesion prevention studies in pelvic surgery in general, Ahmad et al. criticized that exactly these results are generally lacking and, therefore, there is no conclusive evidence that any adhesion prevention devices could lead to respective improvements albeit these would be important [22].

Compared to the results of previous studies cited above, the present study provides data on adhesion prevention in endometriosis surgery and efficacy values that are higher than those of the aforementioned anti-adhesion agents. Notably, 4DryField^®^ PH is not only anti-adhesive but (when applied as a powder) is also marketed as a hemostat. This capability possibly plays a major role for its advantageous adhesion prevention as the basis for adhesion development is the formation of polyfibrin meshes between surgical trauma sites and adjacent tissue. As fibrin is a component of blood, minimizing blood secretion through rapid hemostasis is a cornerstone for adhesion prevention. If conventional agents without an additional hemostatic effect are used on sites with minor but diffuse bleeding, such as those occurring during endometriosis resection, it seems to be more difficult to achieve a peritoneal condition with less fibrin products. Only one of the aforementioned other adhesion prevention agents is a gel. This is noteworthy as according to the instructions for use, a mesh such as Interceed^®^ can take up blood from remaining bleeding sites, which leads to fibrin deposits within the mesh. These fibrin deposits are prone to initiate and promote the formation of adhesions. A solution such as Adept^®^ may dilute the fibrin containing exudates and have an effect by further distancing opposite wound areas. However, the possible distribution of fibrin components might also promote adhesion development in areas where originally no molecular formation trigger is present.

Adhesion formation generally occurs within about 3 to 5 days post-operatively as this is the time until mesothelial healing is completed [35]. An adhesion barrier should remain in place for this period to ensure sufficient separation of wound areas. After that, it should be resorbed as rapidly as possible to avoid any foreign body reactions with potentially negative effects such as local or systemic inflammation. 4DryField^®^ PH is resorbed within 7 days post-operatively. In contrast, Interceed^®^ is absorbed within 4 weeks and Oxiplex/AP^®^ takes even 6 weeks, increasing the potential risk of disadvantageous reactions. On the other hand, the liquid Adept^®^ is already absorbed after 3 to 4 days, which is potentially prior to the complete mesothelial healing.

The degradation and impact on wound healing of 4DryField^®^ PH has been examined in an animal model [36]. The authors found that 7 days after the intervention, the product was almost completely degraded to (poly-)glucose. Histopathologically, *submesothelial* fibrous tissue and mononuclear cells indicated an active healing process with a macroscopically smooth and shiny reconstitution of the visceral and parietal *peritoneal surface*. Additional histopathological analyses performed during an animal study on adhesion prevention [29] showed complete reconstitution of the peritoneum with an intact single layer at the sites of previous abdominal wall and cecal defects after 7 days. Since it is likely that the activity of cellular mediators in the peritoneal fluid plays an active role in peritoneal healing [37], an effective adhesion barrier should not completely prevent the desirable immigration of beneficial cells from the peritoneal fluid. Considering the results from the aforementioned study, 4DryField^®^ PH apparently does not hinder this process and is likely to support mesothelial healing. Comparative examinations of mesothelial healing for the three other barriers are not available.

It has been stated that acute inflammation can enhance adhesion formation mediated through humoral factors in the peritoneal fluid [38]. In animal models, this cascade was decreased by the following parameters: ≥ 5% N_2_O in the pneumoperitoneum [39], cooling of the peritoneal cavity [40, 41], addition of dexamethasone [42], addition of 4% O_2_ to the CO_2_ in the pneumoperitoneum (resulting in 28 mmHg partial oxygen pressure) to correct the mesothelial hypoxia induced when pure CO_2_ is used [43], desiccation prevention [44], and by decreasing blood or fibrin deposits [39].

Of these, the single most important factors are the addition of N_2_O (for which a drug-like effect is assumed but has not yet been found) and cooling (which makes the cells more resistant to trauma by decreasing the metabolism) [45]. In an animal model, the combination of all aforementioned factors (so-called full conditioning) decreased adhesion formation by 76%, if combined with a barrier by even 85% [42]. Specifically, for the resection of deep endometriosis, peritoneal full conditioning in combination with a barrier has reduced adhesion formation in a small clinical RCT [45]. In this aspect, a comprehensive analysis for 4DryField^®^ PH is not available yet, thus it can be speculated how the characteristics interact with the peritoneal surface: supposedly, the gel formulation (intervention group) prevents desiccation and reduces the irritating effect and hypoxia by pure CO_2_; however, saline solution only (control) has also demonstrated a favorable impact on peritoneal conditioning [46, 47] and may reduce adhesion formation after surgical trauma [48, 49]. This appears to be related to altered peritoneal fibrinolytic activity as higher tPA and tPA activity levels were measured [48]. Presumably, this effect is prolonged after 4DryField^®^ PH application as it binds the saline in the gel formulation and holds it back from rapid resorption by the lymphatic system.

Patients were blinded to the group assignment as recommended by Probst et al. [50]. As both treatments in the present study are clearly distinguishable and no placebo for 4DryField^®^ PH exists, the operating surgeon automatically knew the group assignment of each patient when carrying out the treatment, thus preventing double blinding. Additionally, the application of the analyzed agent (4DryField^®^ PH vs. control) in the first surgery had to be repeated in the second intervention according to the study protocol to enable the evaluation of long-term outcomes after the second surgery, which are not a part of this paper. The rationale for this design is that possible adhesions formed after the second intervention would have interfered with the adhesion prevention effects after the first intervention and therefore mislead the interpretation of follow-up results. Despite the general advantages of double blinding, trials which are not double blinded should not automatically be deemed inferior, and proper reporting of the blinding efforts should be considered crucial [51]. In the present study, adhesion scores were taken before the surgeon was unblinded by the study nurse for group-specific treatment at the end of each surgery. Therefore, assessment of adhesion scores was carried out while the operating surgeon and assessor was still blinded. A subsequent evaluation of adhesion scores based only on operative images or videos to enable complete blinding of the assessor was considered inferior to direct intra-operative assessment, as the assessment of adhesion severity is based, among other criteria, on the extent of force required to lyse the adhesion following the AFS score [27]. Such a subtle distinction is hardly possible based on images or videos only. Furthermore, interpretation of images or videos at a later stage carried the risks that not all areas were documented or that different layers of adhesions situated in rows behind each other were not correctly interpreted and scored inaccurately. This particularly applies to the extent score. Another limitation may be the learning curve for the application of the study device. However, it can be speculated that this is a minor aspect as the laparoscopic application via the specially designed application system 4DFLap^TM^ (Fig. 2) is easy to handle.

Yet, the application of various amounts of the agent is an issue: on the treated surfaces at the lesion sites (e.g., cul de sac), an even distribution of the powder is not always possible and certain areas inevitably receive a thicker coverage than others, also leading to a certain degree of variation in the amount of powder applied. In a similar manner, the volume of saline solution used to transform the powder into a gel varies interindividually and between different operative sites. Due to the difficulty of fully standardizing the application, a certain variation in the effectiveness of the product between patients can be anticipated. Furthermore, it is not yet clear if there is an optimal dose and if a thicker layer of the barrier or transformed gel is more effective than a thinner layer. Ahmad and Crescenti [23] described the use of substantial amounts and thicker coverage of 4DryField^® ^PH after visceral adhesiolysis and reported highly effective adhesion prevention. Generally, it has to be investigated for this powder as well as for other products on the market whether the site-specific application of greater amounts of the agents are more important for their anti-adhesive efficacy or if a minimum dose/coverage is sufficient without any further improvement with higher doses.

If no extensive hemostasis is required, 4DryField^® ^PH can be premixed extracorporeally and subsequently be administered as gel. This application has been described in previous publications [23, 24, 26, 29, 31] and might allow a more even distribution of the gel on wound areas and a more standardized ratio between a defined amount of powder and fixed volume of saline solution. The evaluation of 4DryField^®^ PH as a premixed gel versus the intraabdominal powder transformation as well as the comparison of both methods against a control group seems to be of practical interest in future RCTs.

## Conclusion

In this randomized, controlled clinical second-look trial, adhesion formation could be reduced significantly by 85% with the adhesion barrier 4DryField^®^ PH. The outcome is in accordance with experimental studies published previously and suggests a practical benefit for endometriosis surgery. The definition of an optimum dose in relation to the peritoneal defect, the preferred application mode (in-situ gel transformation vs. extracorporeally premixed gel), as well as the evaluation of secondary endpoints are parameters for subsequent investigations. In this regard, the agent’s specific molecular impact on the peritoneal conditions is of interest and warrants further experimental and clinical trials.
